# CCL2/CCR2 and CX3CL1/CX3CR1 chemokine axes and their possible involvement in age-related macular degeneration

**DOI:** 10.1186/1742-2094-7-87

**Published:** 2010-12-02

**Authors:** William Raoul, Constance Auvynet, Serge Camelo, Xavier Guillonneau, Charles Feumi, Christophe Combadière, Florian Sennlaub

**Affiliations:** 1 INSERM, UMR S 872, Centre de Recherche des Cordeliers, F-75006, Paris, France; 2 UPMC Univ Paris 06, UMR S 872, F-75006, Paris, France; 3 Université Paris Descartes, UMR S 872, F-75006, Paris, France; 4 INSERM, UMR_S945, Laboratoire d'Immunologie Cellulaire, F-75013, Paris, France; 5 UPMC Univ Paris 06, UMR_S945, F-75006, Paris, France; 6 AP-HP, Groupe hospitalier Pitié-Salpétrière, Service d'Immunologie, F-75013, Paris, France; 7 AP-HP, Hôtel Dieu, Service d'Ophtalmologie, F-75001, Paris, France

## Abstract

The causes of age-related macular degeneration (AMD) are not well understood. Due to demographic shifts in the industrialized world a growing number of people will develop AMD in the coming decades. To develop treatments it is essential to characterize the disease's pathogenic process. Over the past few years, numerous studies have focused on the role of chemotactic cytokines, also known as chemokines. Certain chemokines, such as CCL2 and CX3CL1, appear to be crucial in subretinal microglia and macrophage accumulation observed in AMD, and participate in the development of retinal degeneration as well as in choroidal neovascularization. This paper reviews the possible implications of CCL2 and CX3CL1 signaling in AMD. Expression patterns, single nucleotide polymorphisms (SNPs) association studies, chemokine and chemokine receptor knockout models are discussed. Future AMD treatments could target chemokines and/or their receptors.

## Introduction

Age-related macular degeneration (AMD) is the leading cause of legal blindness in the developed world [1]. The pathology is characterized by lesions of photoreceptors, retinal pigment epithelium (RPE), Bruch's membrane (BM) and choriocapillaris [2]. Physiologically, the RPE phagocytoses, degrades and recycles photoreceptor outer segments, and clears the debris through the underlying BM into the choroidal circulation. RPE cells selectively transport nutrients from the choroidal capillaries to the outer retina (external hemato-retinal barrier). In the early stages of AMD changes in RPE pigmentation and the excessive presence of yellowish-white subretinal deposits called drusen are clinically observable (fundoscopy) [2,3]. Drusen are composed of lipids and proteins [4], located on the BM, they are partially covered by the RPE. It is believed that drusen are formed because of a transport defect between the RPE and the choriocapillaries or as a result of degenerating RPE cells [5]. There are two clinical forms of late AMD: the fast progressing exudative form defined by choroidal neovascularisation, responsible for the majority of legal blindness in AMD, and the more slow progressing atrophic form characterized by RPE atrophy, photoreceptor degeneration and choroidal involution and obliteration [6,7]. Some of these features can be simulated in a variety of animal models [8], but no animal model has consistently reproduced AMD.

In recent years, there has been increasing evidence for an inflammatory component in AMD. Drusen deposits have been reported to contain immune complexes, complement factors, major histocompatibility complex (MHC) and amyloid oligomers, among others [5,9-12]. A more recent discovery, that AMD is associated with a polymorphism of complement factor H (CfH) [13-15], a polymorphism that leads to an overactivation of the complement system [16,17], emphasizes the importance of inflammatory mediators in AMD. Nevertheless, it remains unclear how the over-activation of the complement system leads to AMD.

Microglial cells (MC) are the resident macrophages of the central nervous system (CNS). In the eye they are only located in the inner retina [18,19]. The subretinal space is physiologically devoid of MCs or macrophages (Mϕ) in healthy young adult subjects. In AMD, MC and Mϕ are activated [9,20] and accumulate in the subretinal space [19,21]. Interestingly, activated complement fraction C3 and C5 participate in neutrophil and macrophage recruitment to the subretinal space in a choroidal neovascularization model [22]. A similar mechanism might participate in retinal/choroidal inflammation in AMD. The prolonged presence of MC/Mϕ in the subretinal space is associated with photoreceptor degeneration [19,23] and the development of choroidal neovascularization in animal models [24,25]. This is possibly because MC/Mϕ are an important source of cytokines and angiogenic factors like VEGF [26,27]. Interfering with subretinal MC/Mϕ accumulation might therefore be a promising avenue in the treatment of AMD, but the mechanisms of subretinal MC/Mϕ cell accumulation remain unclear.

Chemokines constitute a family of structurally related chemotactic cytokines that direct the migration of leukocytes throughout the body, both under physiological and pathological conditions [28]. CCL2 signaling through CCR2, and CX3CL1 signaling through CX3CR1 are key factors in Mϕ recruitment to a tissue lesion [29,30]. In this review, we discuss the possible role of CCL2/CCR2 and CX3CL1/CX3CR1 axes in MC/Mϕ homeostasis in the healthy eye and in AMD. We review data describing the expression of these chemokines and their receptors in the retina, polymorphism studies in AMD, animal chemokine and chemokine receptor knockout models presenting drusen formation, and photoreceptor degeneration or choroidal neovascularisation.

### CX3CL1, CX3CR1, CCR2 and CCL2 expression in the retina

In the blood, chemokine receptors CCR2 and CX3CR1 identify two functional subsets of murine blood monocytes: "inflammatory" monocytes, which express both receptors, and non-inflammatory monocytes, which only express CX3CR1 [31]. CCL2 and CX3CL1 released by tissue lesions participate in the recruitment of monocytes and in local inflammation [29,30].

CX3CL1 is an atypical chemokine. It is expressed as a transmembrane protein, which can be cleaved by proteases into a soluble form that has chemotactic properties [32]. In its transmembrane form, CX3CL1 mediates integrine-like intracellular adhesion. Unlike many promiscuous chemokines, it only signals through the CX3CR1 receptor [33]. In the eye, it is constitutively expressed in retinal neurons and in the RPE [34] CX3CL1 can also be induced in microvascular endothelial cells [34]. In the retina, the vast majority of resident "quiescent" MCs express CX3CR1 in newborn (PN6) and adult mice (6 to 18 months) [19,35]. Immunohistochemistry reveals similar results in humans [19]. Contrary to a previous report, the use of CX3CR1-specific antibody in humans and experiments with Cx3cr1^GFP/+ ^mice [36] failed to find significant CX3CR1 expression in RPE cells *in vivo *[37]. MCs are the only cells in the retina that express CX3CR1 under physiological conditions.

CCL2 expression in the retina and RPE is very low in healthy young adult animals [38], but increases in acute inflammation [23,39,40], with aging [38] and under oxidative stress in the RPE [41].

Recent evidence suggests that subretinal MC/Mϕ induce CCL2 and CCL5 in the RPE [42]. CCL2 mainly signals through CCR2 [43]. There is no direct evidence of CCR2 expression by retinal MCs or macrophages in the retina. However, it has been shown that CCL2/CCR2 signaling is involved in monocyte or MC recruitment after laser injury [44,45] and aging [45] in knockout mice. This suggests that CCR2-expressing monocytes or MCs are present at some point in these models. In the brain, CCR2 expression has been reported to be very low in healthy rat CNS microglia, but large numbers of CCR2 positive MC/Mϕ are found in acute inflammation [46]. To summarize, CX3CL1 and CX3CR1 are constitutively and robustly expressed in the retina and might have a role in retinal homeostasis. In contrast, CCL2 is expressed at low levels in the healthy young adult, but increases with age and injury. A very recent clinical study shows that elevated intraocular CCL2 levels are associated with exudative AMD [47]. CCL2 might therefore play a role in monocyte and microglial cell recruitment to the subretinal space with age and in AMD.

### *CX3CR1*, *CCR2 *and CCL2 single nucleotide polymorphisms (SNPs) AMD

Several studies have examined SNPs of the chemokine system and AMD susceptibility. The T280M allele of the *CX3CR1 *gene has been shown to be associated with AMD in a group of Caucasian patients recruited in the Washington D.C. area [37]. We replicated this association in a group of C-aucasian patients recruited in Paris [19] and a similar association has recently been found in Han Chinese patients recruited in Nantong [48]. Previous studies show that the M280 polymorphism provokes loss of chemotaxis [49] or increases adherence to its ligand [50]. Therefore, dysfunctional CX3CL1/CX3CR1 signaling might play a role in AMD (see below). No evidence was found of an association between common genetic variations of CCR2, and CCL2 and AMD [51].

### The *Cx3cr1*^-/- ^mouse model of AMD

As the T280M allele of the CX3CR1 leads to dysfunctional monocyte migration [19] and is associated with AMD [19,37,48], several groups have studied Cx3cr1^-/- ^mice to decipher the effect of CX3CR1 dysfunction on ocular homeostasis. As with monocytes bearing the T280M allele, Cx3cr1^-/- ^MCs display inhibited migration [52] when compared with CX3CR1 competent MCs. We have shown that Cx3cr1^-/- ^mice spontaneously accumulate subretinal MC/Mϕ with age in the absence of photoreceptor degeneration [19]. Furthermore, an increased accumulation of subretinal MC/Mϕ is found after light injury in comparison to a wildtype control group [53]. These results stand in sharp contrast to observations of inflamed peripheral tissue, where Cx3cr1^-/- ^mice similar to other chemokine receptor knockouts present an inhibition of Mϕ accumulation [30].

It has since been recognized that subretinal MC/Mϕ also accumulate in wild type mice at a more advanced age [54], suggesting that dysfunction of certain chemokines and possible other factors accelerates a physiological process leading to MC/Mϕ accumulation at an earlier age in Cx3cr1^-/- ^mice as compared to controls. Ng *et al. *[55] showed that normal animal facility lighting conditions induced subretinal MC/Mϕ accumulation in the absence of photoreceptor degeneration in albino strains. Furthermore they show that subretinal MC/Mϕs are cleared from the subretinal space when light stimulus is removed. These observations suggest that MC/Mϕs migrate to the subretinal space and are subsequently cleared once the stimulus is removed in the absence of primary pathological photoreceptor or RPE lesions. It remains unclear if this clearance is due to apoptosis of the subretinal MC/Mϕ or to migration from the subretinal space. In retinal degeneration, where severe degeneration occurs, we observed an egress of rhodopsin laden MC/Mϕs from the subretinal space [53], suggesting that MC/Mϕs can leave the subretinal space by migration. The migratory deficit observed in Cx3cr1^-/- ^microglia [52] might contribute to the reduced clearance from the subretinal space and therefore accelerate the accumulation, as observed in aged Cx3cr1^-/- ^mice. Nevertheless, it cannot be excluded that reduced subretinal MC/Mϕ apoptosis plays a role in subretinal MC/Mϕ accumulation.

In Cx3cr1^-/- ^mice, the resulting prolonged presence of subretinal MC/Mϕ in the subretinal space is associated with excessive OS phagocytosis by the MC/Mϕs, which subsequently ingest intracellular lipids [19,56]. These subretinal MC/Mϕ "foam cells" are the origin of the drusen-like deposits observed in the clinical observation of Cx3cr1^-/-^mice [19] and have recently been reported in Ccl2^-/- ^mice [45] (see below). Similarly, CX3CR1-positive bloated subretinal microglial cells are found in the eyes of AMD patients [19,21]. Recent reports suggest that drusen appearance in patients with AMD is not solely caused by sub-RPE deposits, but also by subretinal drusenoid deposits [57-59] not unlike the lesions observed in Cx3cr1^-/- ^and Ccl2^-/- ^mice. It is tempting to speculate that these drusenoid deposits are the consequence of accumulated debris from subretinal MC/Mϕ "foam cell" apoptosis. In consequence, drusen might evolve from subretinal deposits which are subsequently covered by the RPE. There are several lines of evidence supporting this hypothesis: drusen contain numerous degenerating organelles, the origin of which may be subretinal MC/Mϕ [4]. Moreover, drusen contain CX3CR1, apolipoprotein E, complement factors, and the major histocompatibility complex (MHC) [5,9-12,19], all of which can be expressed by MC/Mϕ [19,60-62]. The debris and inflammatory proteins found in drusen may originate in part from MC/Mϕ additionally to RPE debris.

Another consequence of the prolonged presence of Cx3cr1^-/- ^MCs in the subretinal space is photoreceptor cell death [19,53] and changes in RPE structure and distribution [42]. In Cx3cr1^-/- ^mice on a pigmented C57Bl6 background, photoreceptor degeneration of about 25-30% is observed in 18-month-old mice as compared to wild type C57BL6 mice and can be induced in two-month-old Cx3cr1^-/- ^mice by exposure to 100 Klux for 10 min, which does not provoke degeneration in wild type C57BL6 mice [19,53]. Cx3cr1^-/- ^mice on an albino Balb genetic background develop complete light-dependent photoreceptor degeneration by the age of four months in 12 h/24 h 100-500 lux (normal animal facility conditions). In a light-induced model that provokes near complete degeneration in pigmented C57Bl6 wild type animals this difference is no longer observed [63], suggesting that a maximal MC/Mϕ cell toxicity can be reached in wild type animals. Activated MC toxicity has been shown in photoreceptors *in vitro *[64] and *in vivo *[23]. Neuronal cell toxicity caused by the prolonged presence of activated Cx3cr1^-/- ^MCs in the brain has been described as a mechanism of neurodegenerative diseases [65]. Similar mechanisms may cause the degeneration observed in the Cx3cr1^-/- ^mice.

Additionally, Cx3cr1^-/- ^mice develop increased MC/Mϕ accumulation and choroidal neovascularization in comparison to controls in a laser-induced choroidal neovascularization (CNV) model, suggesting that the presence of subretinal MC/Mϕs contributes to a proangiogenic environment [19]. MC/Mϕs might produce proangiogenic factors themselves, but more importantly, they have been shown to induce MMP9 and VEGF expression in the RPE [42]. This may help to explain the increase in CNV observed in Cx3cr1^-/- ^mice.

In summary, Cx3cr1^-/- ^mice develop primary subretinal MC accumulation, possibly resulting from a migratory defect associated with CX3CR1 dysfunction. We have shown that the M280 polymorphism of CX3CR1, associated with AMD increases adherence to its ligand [50]. The increased adherence to transmembraneous CX3CL1 abundantly present in the retina and RPE [34] significantly inhibits the mobility of monocytes expressing the CX3CR1-M280 variant [19]. If similar alterations occur *in vivo *to MC/Mϕ, the M280 polymorphism may cause excessive MC/Mϕ adherence to membrane-anchored CX3CL1 in the retina and RPE [34] and reduce migration in response to other inflammatory chemoattractants. In subjects with the M280 polymorphism, clearance of MC/Mϕs from the subretinal space (in response to soluble CX3CL1 or other chemoattractants) would thereby be inhibited, and subretinal MC/Mϕ accumulation might occur.

The prolonged presence of subretinal MC/Mϕs could thereby lead to subretinal drusenoid deposits, retinal and RPE degeneration, and an increase in CNV as observed in Cx3cr1^-/- ^mice. These results suggest that MC/Mϕ accumulation in the subretinal space may be a driving force in the pathogenesis of AMD and not a mere consequence of primary RPE or photoreceptor disease.

### C*cl2*^-/- ^*and *C*cr2*^-/- ^mouse models of AMD

There are several reports using Ccl2^-/- ^or Ccr2^-/- ^mice in an attempt to decipher the inflammatory mechanisms of AMD. CCL2 is increased intraocularly in exudative AMD [47] and in a mouse model of choroidal neovascularization [40]. Tsutsumi *et al. *[44] reported that macrophages extracted from eyes undergoing the laser-induced CNV model are angiogenic and that the recruitment of MC/Mϕs to the injury site and subsequent CNV are reduced in Ccr2^-/- ^mice. These results have since been corroborated in Ccl2^-/- ^mice [45]. Supporting this data is the repeated observation that macrophage depletion inhibits CNV [24,66]. In contrast, Ambati *et al. *observed spontaneous CNV in 4 of 15 Ccl2^-/- ^and 3 in 13 Ccr2^-/- ^mice older than 18 months of age, identified by angiographic leakage. Luhmann *et al. *were unable to detect spontaneous CNV by angiography or immunohistochemistry in 11 Ccl2^-/- ^mice aged 16 to 25 months [45], suggesting that CCL2 deficiency is not sufficient to induce spontaneous neovascularization. Ambati *et al. *also observed the spontaneous appearance of yellowish white subretinal deposits in fundoscopies of Ccl2^-/- ^and Ccr2^-/- ^mice aged 9 months and older, which they referred to as "drusen". The authors suggested that the deficiency in Mϕ recruitment through a CCL2-/CCR2-dependent pathway from choroidal circulation may prevent the clearance of accumulating debris in BM [25], which, over time, would lead to drusen formation. Luhmann et al. corroborated the spontaneous appearance of funduscopically observed autofluorescent lesions that resemble drusen in 16- to 25-month-old Ccl2^-/- ^mice. However, these lesions are not caused by the sub-RPE extracellular deposits believed to be the origin of drusen appearance in humans. In fact, the anatomical equivalent of the lesions observed in Ccl2^-/- ^mice was found to be bloated subretinal lipid MC/Mϕs detectable by immunohistochemistry [45], similar to those described in Cx3cr1^-/- ^mice [19,56]. It is difficult to appreciate what structure led to the drusen-like lesions described by Ambati et al. [25] because immunohistochemical analysis of subretinal MC/Mϕs were not performed and no histological evidence of typical convex shaped local BM deposits are shown.

In terms of photoreceptor degeneration Ambati *et al. *[25], using electron microscopy, observed pyknotic photoreceptor cell nuclei in 16-month-old Ccl2^-/- ^mice but not in wild type animals. Nevertheless, the quantification of photoreceptor in 23- to 25-month-old mice performed by Luhmann *et al. *[45] showed no significant degeneration in Ccl2^-/- ^mice as compared to wild type congeners, suggesting that Ccl2 invalidation does not lead to significant photoreceptor degeneration.

The conclusion drawn from Ambati's data [25], that AMD develops because of a CCR2-dependent macrophage recruitment deficit and therefore a hypoinflammatory state, is in contradiction with a recent report of increased intraocular CCL2 levels in AMD [47] and abundant evidence of MC/Mϕ accumulation in AMD [9,19-21]. Further studies are needed to identify the additional factors that led to the discrepancy of Luhmann *et al.*'s and Ambati *et al.*'s results.

### *The Ccl2*^-/-^*Cx3cr1*^-/- ^mouse model of AMD

Tuo *et al. *[37] were the first to describe an association between the T280M Cx3cr1 allele and AMD in 2004. Shortly thereafter, Ambati et al. [25] reported that Ccl2^-/- ^and Ccr2^-/- ^mice develop AMD-like features at an advanced age. In an attempt to accelerate the development of AMD-like features, Tuo *et al. *[67] generated Ccl2^-/-^Cx3cr1^-/- ^mice that indeed develop "drusen," pigment alterations, and retinal degeneration by the age of 6 weeks in 100% of mice. Nevertheless, independently generated Ccl2^-/-^Cx3cr1^-/- ^mice in our laboratory do not present any of these features at 6 weeks of age, and are indistinguishable from Ccl2^-/-^, Cx3cr1^-/- ^and wild type animals. Tuo and Chan [68] also reported an abnormal mendelian segregation of the Ccl2^-/-^Cx3cr1^-/- ^genotype, poor reproduction, and progressive patchy skin depigmentation, all of which we have not encountered in the generation of our Ccl2^-/-^Cx3cr1^-/- ^mice [29]. Tuo *et al. *[68] selected the founding breeding pair of their Ccl2^-/-^Cx3cr1^-/- ^mice "with the most retinal drusen-like lesions". By selecting the founding breeding pair for AMD-like features, the authors may have selected animals genetically predisposed to these lesions independent of their Ccl2 or Cx3cr1 invalidation. The non-reproducibility of the ocular and systemic features in our Ccl2^-/-^Cx3cr1^-/- ^mice, in which the breeding pairs were not selected for any feature other than the genetic invalidation of Ccl2 and Cx3cr1, suggests this is the case. The conclusions concerning the implication of CCL2 in the phenotype of this mouse strain should therefore be taken with caution.

## Conclusions and perspectives

CX3CL1 and CX3CR1 are robustly expressed in the healthy retina. Their dysfunction leads to subretinal MC/Mϕ accumulation with deleterious effects to the RPE and photoreceptors. The association of AMD with a polymorphism in the Cx3cr1 gene, leading to a dysfunctional CX3CR1 protein, and the observation that MC/Mϕs accumulate in the subretinal space in AMD suggests that dysfunctional CX3CL1/CX3CR1 signaling might play a role in the pathogenesis of AMD. However, the mechanism that leads to the MC/Mϕ accumulation remains obscure and further research is needed to identify the implicated actors.

In contrast, CCL2 and CCR2 expression in the healthy retina is low. Injuries, such as laser impacts or retinal detachment, lead to a CCL2/CCR2 dependent recruitment of MC/Mϕ to the subretinal space with deleterious effects to the photoreceptors and to the progression of CNV [23,44]. A recent clinical study that shows an association of increased intraocular CCL2 levels and AMD [47] supports the possible involvement of CCL2 in the pathogenesis of AMD. It is not well understood if CCR2 is expressed in the healthy retina. The observation that Ccl2^-/- ^mice also develop subretinal MC/Mϕ accumulation at a later stage might indicate an age-dependent shift in populations of CX3CR1 + CCR2- microglia to CX3CR1 + CCR2 + microglia and that at this later stage, CCL2/CCR2 signaling is implicated in the clearance of subretinal MC/Mϕs similar to the phenomenon observed in Cx3cr1^-/- ^mice. These results would suggest that the inhibition of CCL2/CCR2 signaling might have a beneficial effect on CNV formation and MC/Mϕ associated photoreceptor degeneration at an early stage of AMD. More research is needed to determine if CCL2/CCR2 inhibition is beneficial at a later stage.

## Competing interests

The authors declare that they have no competing interests.

## Authors' contributions

WR, CA, SC, XG, CF and CC contributed to the writing of different sections of the manuscript. FS wrote the manuscript. All authors have read and approved the final version of the manuscript.
